# Cotton-textile-enabled flexible self-sustaining power packs via roll-to-roll fabrication

**DOI:** 10.1038/ncomms11586

**Published:** 2016-05-18

**Authors:** Zan Gao, Clifton Bumgardner, Ningning Song, Yunya Zhang, Jingjing Li, Xiaodong Li

**Affiliations:** 1 Department of Mechanical and Aerospace Engineering, University of Virginia, 122 Engineer's Way, Charlottesville, Virginia 22904-4746, USA; 2 Department of Mechanical Engineering, University of Hawaii at Manoa, 2540 Dole Street, Honolulu, Hawaii 96822, USA

## Abstract

With rising energy concerns, efficient energy conversion and storage devices are required to provide a sustainable, green energy supply. Solar cells hold promise as energy conversion devices due to their utilization of readily accessible solar energy; however, the output of solar cells can be non-continuous and unstable. Therefore, it is necessary to combine solar cells with compatible energy storage devices to realize a stable power supply. To this end, supercapacitors, highly efficient energy storage devices, can be integrated with solar cells to mitigate the power fluctuations. Here, we report on the development of a solar cell-supercapacitor hybrid device as a solution to this energy requirement. A high-performance, cotton-textile-enabled asymmetric supercapacitor is integrated with a flexible solar cell via a scalable roll-to-roll manufacturing approach to fabricate a self-sustaining power pack, demonstrating its potential to continuously power future electronic devices.

## Abstract (web summary)

Activated cotton textiles are promising for wearable energy storage due to their flexibility and high conductivity, but scalability is an issue. Here, the authors combine supercapacitors and solar cells on activated cotton textiles via scalable roll-to-roll assembly to make self-sustaining power packs.

## Graphical abstract

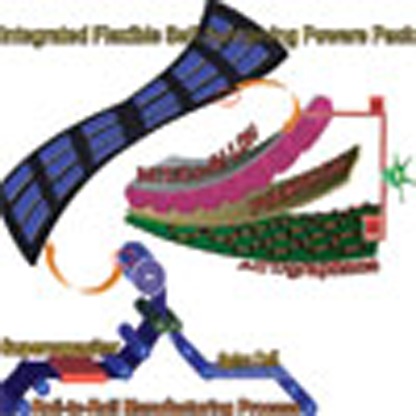

With ever-increasing global energy consumption and the depletion of fossil fuels, finding a sustainable and clean energy supply has become one of the most important scientific and technological challenges facing humanity today^1^. Fossil fuels such as coal, oil and natural gas are limited and nonrenewable. With growing energy demand in many urbanizing nations, particularly in India and China, the threat of depleting the planet's fossil fuel reserves is of increasing concern. Relief may be as simple as utilizing the most accessible solar energy. Only recently, thanks to the development of less expensive and more efficient photovoltaic cells, solar energy has been harnessed and widely used as an alternative energy source. However, due to the fluctuation of light intensity and the diurnal cycle, the output of a solar cell can be non-continuous and unstable. Therefore, there is a clear need to combine solar cells with energy storage devices (serving as a buffer) to mitigate the power fluctuations and to allow operation of the cell and storage device as a reliable source of energy. Among various emerging energy storage technologies, supercapacitors have been regarded as a very promising energy source for next-generation electronics and electric vehicles because of their excellent properties, such as high power density, long lifespan, environment benignancy, safety and low maintenance cost^2^.

Compared with lithium-ion batteries, the relatively low energy density of supercapacitors limits their wide application. Developing new electrode materials with well-defined nano-architecture and high active surface area is key to improving the electrochemical performance of supercapacitors. To date, according to the different mechanisms of charge storage, typical supercapacitor electrode materials can be categorized into electric double-layer capacitive materials including various carbon materials^3^,^4^ and pseudo-capacitive materials including transition metal oxides^5^,^6^,^7^,^8^, and conductive polymers^9^,^10^,^11^. However, different materials have different advantages and disadvantages for supercapacitor applications. For example, carbon materials usually possess higher power density and longer lifespans but lower energy density. Transition metal oxides/hydroxides and conductive polymers possess higher energy density but poorer cyclic life and lower power density. One intelligent strategy to strengthen the electrochemical performance of supercapacitors is to develop a hybrid material of carbon and metal oxides/hydroxides, which is anticipated to jointly improve the overall performance of supercapacitors in terms of their energy density, power density and cyclic stability^12^.

Alternatively, a more attractive approach for balancing the contradiction between energy density and power density of supercapacitors is to assemble an asymmetric supercapacitor, which consists of a battery-type Faradic electrode as energy source (usually pseudo-capacitive materials) and a capacitor-type electrode as power source (usually carbon materials)^13^,^14^. In the last few decades, metallic layered double hydroxides (LDHs) with the general chemical formula [M^II^_1-*x*_M^III^_*x*_(OH)_2_]^*x*+^[A^*n*−^]_*x/n*_·*m*H_2_O, where M^II^ and M^III^ are divalent and trivalent metal cations and A^*n*−^ are the charge-balancing anions^15^, have aroused great interest in catalysis^16^, biotechnology^17^, separation^18^ and electrochemistry^19^,^20^. Moreover, LDHs have been proven to be a promising class of electrode materials for supercapacitors because of their relatively low cost, high redox activity and environmentally friendliness^21^,^22^. For example, Huang *et al.* reported that nickel-aluminium LDH (Ni-Al LDH) deposited on nickel foam exhibits an ultrahigh specific capacitance of 2,123 F g^−1^ at 1 A g^−1^ (ref. 23). Chen *et al.* also demonstrated that nickel-cobalt LDH (Ni-Co LDH) film had a significantly enhanced specific capacitance of 2,682 F g^−1^ at 3 A g^−1^ (ref. 24). In our previous work, Ni-Al LDH was coupled with graphene nanosheets to prepare a hybrid electrode, which also demonstrated excellent electrochemical performance^25^. Therefore, high-energy LDHs are ideal candidates for asymmetric supercapacitors. On the other hand, two-dimensional (2D) monolayered graphene has been intensively explored for energy storage application because of its superior electrical conductivity, high specific surface area, outstanding mechanical properties and relative wide operation windows^26^,^27^,^28^,^29^. Graphene-based films and papers have been shown to be ideal ‘power sources' for flexible, asymmetric supercapacitors^30^,^31^,^32^,^33^.

Consumer demand for portable/wearable electronics has triggered a technological race to drive innovation in flexible mobile phones, bendable displays, electronic skin and distributed sensors^34^,^35^,^36^. Many attempts have been devoted to developing safe, lightweight and flexible power sources to meet the urgent demand for flexible and wearable electronics^37^,^38^,^39^,^40^. In addition, ‘self-powered nanotechnology' has been proposed to enable future electronics to operate independently and sustainably without batteries, or with a battery possessing extended lifespan^41^. Self-powered nanosystems with multi-functionalities will be a prominent driving force for future world economies by employing transformative nanomaterials and nanofabrication technologies^42^. To date, many attempts and achievements have been made in developing self-powered nanosystems by combining nanogenerators or solar cells with lithium-ion batteries or supercapacitors^43^,^44^,^45^. However, until now, a streamlined manufacturing process for integrating a flexible energy harvesting unit with a flexible energy storage unit has not been achieved due to the lack of effective packaging technology.

Cotton textile, a source of flexible, ‘green', renewable, breathable clothing, has been shown to be an excellent wearable platform for constructing flexible energy storage devices as activated cotton textiles (ACTs) exhibit eminent flexibility and excellent conductivity^46^,^47^. In this work, flower-like cobalt-aluminium LDH (Co-Al LDH) nanoarrays consisting of interconnected nano-petals and pompon-like nano-stamens are anchored *in situ* on flexible ACTs to realize ACT/Co-Al LDH hybrid by a facile hydrothermal method. A highly conductive graphene coating is wrapped around ACT fibres to form ACT/graphene composite by a simple dipping, drying and reducing process. A flexible, all-solid-state asymmetric supercapacitor (ACT/Co-Al LDH//ACT/graphene) is assembled using the nanostructured ACT/Co-Al LDH as the positive electrode, ACT/graphene as the negative electrode, and PVA-KOH gel as both the solid-state electrolyte and separator. The asymmetric cell works synergically to achieve excellent electrochemical performance in terms of its working potential (1.6 V), energy density (55.04 Wh kg^−1^), power density (5.44 kW kg^−1^) and cycling stability (capacitance retention rate of 87.54% after 2,000 cycles). Moreover, we demonstrate a practical roll-to-roll manufacturing approach to combine the flexible asymmetric supercapacitor with a flexible solar cell to fabricate an integrated self-sustaining power pack, which is scalable for industrial manufacturing. Importantly, the assembled power pack can power a commercial light-emitting-diode (LED) continuously, with or without sunlight, demonstrating its potential for flexible, self-powered energy devices.

## Results

### Positive electrode materials

**Figure 1:**
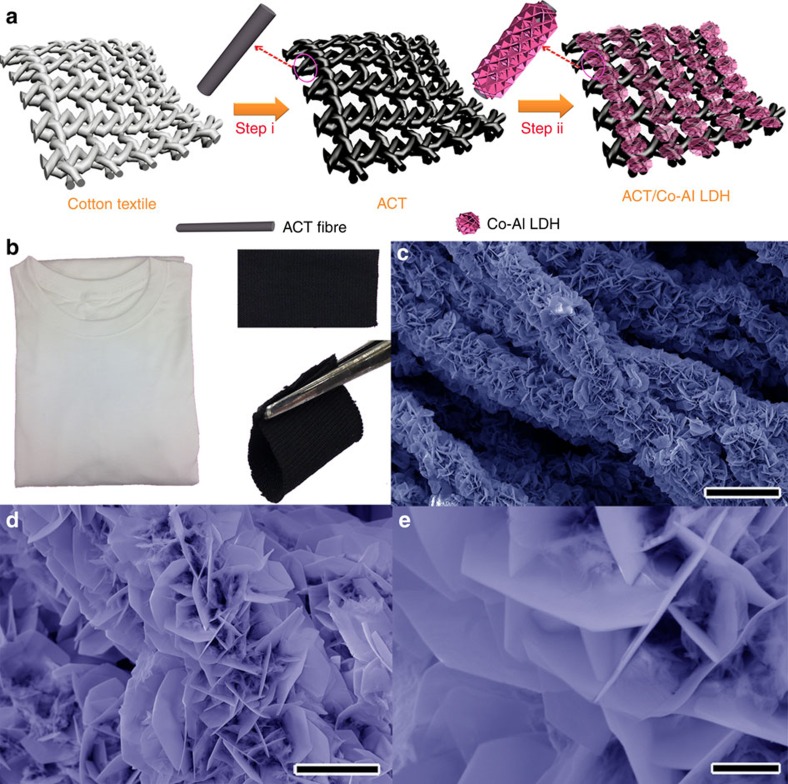
Synthesis and characterization of ACT/Co-Al LDH composite. (**a**) Schematic illustration of the formation processes of Co-Al LDH nanostructure on the ACT. (**b**) Photographs of a cotton T-shirt, a piece of ACT and a piece of ACT under folded state. (**c**–**e**) SEM images of Co-Al LDH nanosheets on ACT fibres at different magnifications. Scale bars, 20 μm for **c**, 5 μm for **d** and 1 μm for **e**.

Figure 1a illustrates the two-step design and synthesis procedures of Co-Al LDH nanoarrays on ACT fibres. In a typical experiment, highly conductive and flexible ACTs were first prepared by direct conversion of cotton T-shirt textiles through a dipping, drying and annealing process (step i). Then, flower-like Co-Al LDH nanoarrays were radially grown *in situ* on ACT fibres using a facile hydrothermal method (step ii), which resulted in a pink coating on the surface of ACT fibres. Figure 1b shows the digital photographs of the cotton T-shirt and the converted ACT under normal and folded states, demonstrating its mechanical flexibility. Figure 1c–e show the scanning electron microscopy (SEM) images of the as-synthesized Co-Al LDH nanoarrays on ACT fibres. High-density hexagonal Co-Al LDH nanosheets, with the length of about 2–5 μm and thickness of around 40–70 nm, were radially aligned on individual ACT fibres, forming flower-like nanoarrays that consist of interconnected LDH nano-petals and pompon-like LDH nano-stamens. Such three-dimensional (3D) hierarchical, porous nanostructure *in situ* anchored on ACT fibres not only enhances the contact between the active material and ACT substrate but also serves as a reservoir for electrolyte ions, shortening ion diffusion path and facilitating charge transfer, jointly enhancing the electrochemical performance.

**Figure 2:**
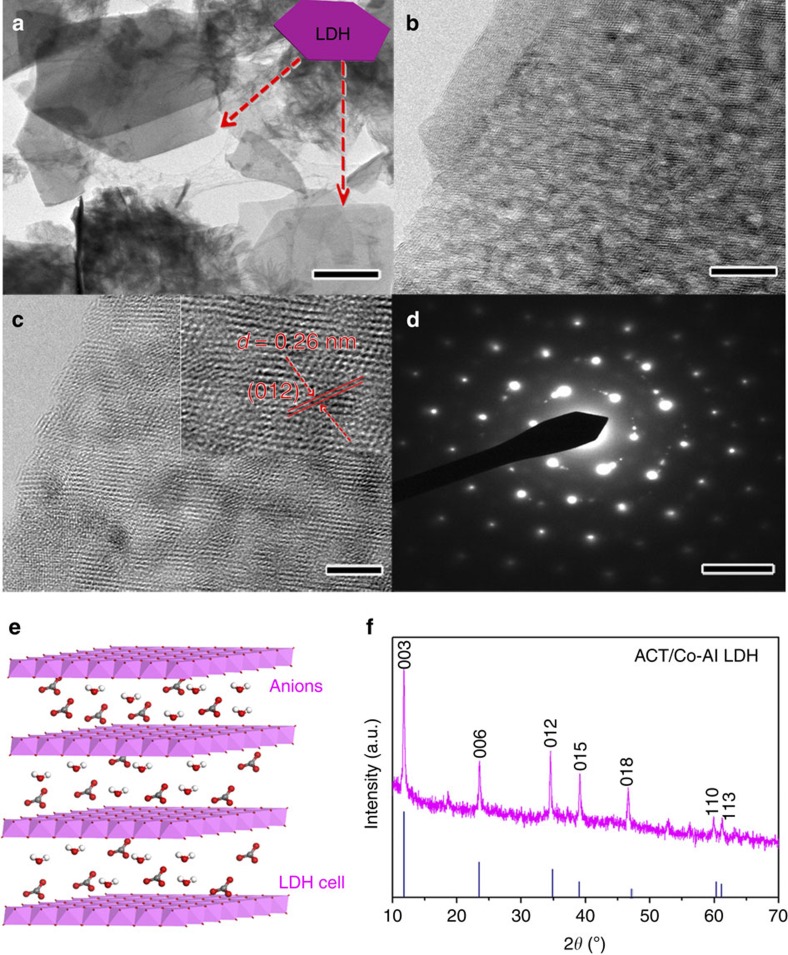
Characterization of the as-obtained ACT/Co-Al LDH composite. (**a**) TEM image of Co-Al LDH (scale bar, 1 μm), inset is the illustration of LDH. (**b**–**d**) HRTEM images of Co-Al LDH and the corresponding SAED pattern. Scale bars, 10 nm for **b**, 5 nm for **c** and 5 nm for **d**. (**e**) Schematic illustration of the molecular structure of Co-Al LDH. (**f**) XRD patterns of ACT/Co-Al LDH and the standard peaks of Co-Al LDH (JCPDS # 510045).

The structure and morphology of the Co-Al LDH nanosheets were further investigated by transmission electron microscopy (TEM) and X-ray diffraction (XRD). TEM inspection (Fig. 2a) reveals that the LDH nanosheets are of hexagonal shape with 2- to 5-nm-sized nanopores on the surface of individual LDH nanosheets (Fig. 2b), which is expected to have a higher specific area. The ACT/Co-Al LDH composite achieved a high Brunauer–Emmett–Teller (BET) surface area of 814.4 m^2^ g^−1^. A lattice spacing of 0.26 nm (Fig. 2c) can be ascribed to the (012) crystal plane of Co-Al LDH, which is in consistent with the XRD results (Fig. 2f). The corresponding selected area electron diffraction (SAED) pattern (Fig. 2d) shows hexagonally arranged bright spots, indicating the single-crystal nature of LDH nanosheets. The XRD pattern (Fig. 2f) shows a series of diffraction peaks at 11.59°, 23.26°, 34.56°, 38.78° and 46.7°, which can be indexed to the (003), (006), (012), (015) and (018) planes of the layered LDH phase (JCPDS #51-0045), respectively. The small peak at ∼19° probably resulted from cobalt hydroxide impurity in the ACT/Co-Al LDH composite.

### Negative electrode material

**Figure 3:**
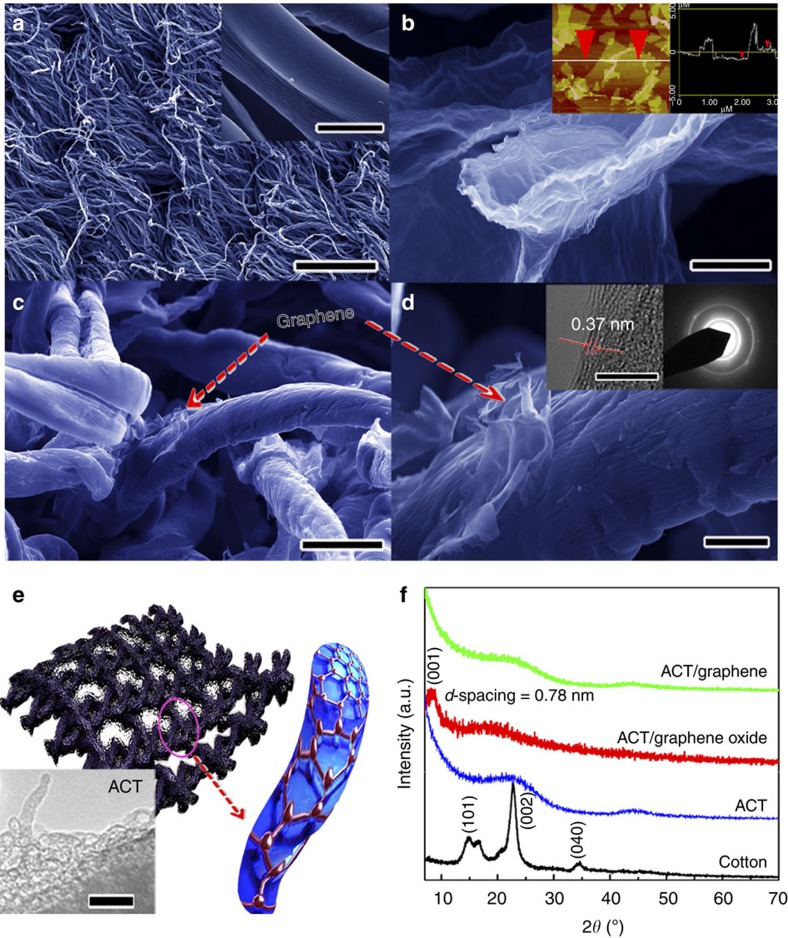
Characterization of the as-obtained ACT/graphene composite. (**a**) SEM image of ACT (scale bar, 250 μm), inset is the amplified SEM image (scale bar, 5 μm). (**b**) SEM image of graphene oxide, inset is the atomic force microscopy image of graphene oxide nanosheets (with a height profile showing a step of 1.1 nm marked by the red arrows). (**c**,**d**) SEM images of ACT/graphene composite at different magnifications (scale bars, 10 μm for **c** and 2 μm for **d**), insets of **d** are the HRTEM image (scale bar, 5 nm) and corresponding SAED pattern of graphene nanosheets. (**e**) Schematic illustration of the ACT fibre with graphene coating, inset is the TEM image of ACT fibre (scale bar, 25 nm). (**f**) XRD patterns of pure cotton textile, ACT, ACT/graphene oxide and ACT/graphene composite.

Highly conductive graphene was coated on flexible ACTs to fabricate negative electrode material for constructing asymmetric supercapacitors. Conductive ACTs (sheet resistance: ∼10–20 Ω sq.^−1^) were first prepared by direct conversion of a cotton T-shirt. As shown in Fig. 3a, the ACT fibres have diameters ranging from 5 to 10 μm, which inherit the cellulose fibre structure of the cotton textile. After activation, a piece of ACT was dipped into graphene oxide solution for a few minutes to coat graphene oxide nanosheets. Figure 3b shows the typical SEM images of the corrugated and scrolled graphene oxide sheets, resembling crumpled silk veil waves. The thickness of the as-synthesized graphene oxide sheets was measured to be 1.1 nm (inset of Fig. 3b), which is close to the theoretical value of 0.78 nm for single-layer graphene oxide, indicating that the as-prepared graphene oxide sheets were predominantly in monolayered manner^48^. Usually, the graphene oxide sheets tend to agglomerate due to the electrostatic interactions of the oxygen-containing functional groups (epoxide, hydroxyl, carbonyl and carboxyl groups) on the surfaces and the edges of the sheets (inset of Fig. 3b). After dipping, individual ACT fibres were wrapped with curled and entangled graphene oxide sheets (a mixture of multilayered/monolayered sheets). During the thermal reduction, the conductivity of the graphene oxide sheets was enhanced due to partial overlapping or coalescing via *π*–*π* stacking or hydrogen bonding^49^,^50^, resulting in an interconnected 3D conductive graphene network on the ACT fibres (Fig. 3c–e). The insets of Fig. 3d are the high-resolution transmission electron microscopy (HRTEM) image and corresponding SAED pattern of the graphene nanosheets. The deformed crystal fringes with a *d*-spacing of ∼0.37 nm in the HRTEM image suggest the existence of defects in the graphene nanosheets. The crystalline nature of the graphene nanosheets was validated by the well-defined diffraction spots in the SAED pattern. More importantly, the ACT fibres showed a porous structure as revealed by the TEM image (inset of Fig. 3e). The entangled graphene sheets wrapped around the porous ACT fibres, forming a 3D porous conductive net. The BET surface area of the ACT/graphene composite was measured to be ∼450 m^2^ g^−1^. The 3D graphene/fibre conductive net is expected to facilitate electrolyte ion diffusion and electron transport in the charge/discharge processes, improving the overall power density of the asymmetric cell.

Figure 3f shows the XRD spectra of cotton, ACT, ACT/graphene oxide and ACT/graphene composites, respectively. The diffraction peaks at 14.8°, 22.7° and 34.3° can be indexed, respectively, to the (101), (002) and (040) peaks of the cellulose polymorphs from Cellulose I ingredients in pure cotton^14^,^51^. After activation, the cotton peaks disappeared, alternately, a broad diffraction peak appeared at ∼21° for ACT, indicating the breakage of cellulose polymorphs and the formation of amorphous carbon during the activation process. The peak at 8.8° for ACT/graphene oxide corresponds to the (001) lattice plane of graphene oxide with an interplanar spacing of 0.78 nm, indicating the complete exfoliation of graphite to graphene oxide^52^. After the thermal reduction process, the peak at 8.8° for the ACT/graphene oxide disappeared, suggesting the oxygen-containing groups on the graphene oxide had been removed, and the conductivity of graphene oxide was restored^53^,^54^, ensuring improved electrochemical performance of the assembled asymmetric cell. Raman spectroscopy (Supplementary Fig. 1) was used to further characterize the ACT/graphene composite (reduced from ACT/graphene oxide). Generally, graphene exhibits two characteristic bands in its Raman spectrum: the G band at ∼1,575 cm^−1^ resulting from the first-order scattering of the E_1g_ phonon of sp^2^ C atoms and the D band at ∼1,350 cm^−1^ resulting from a breathing mode of point photons of A_1g_ symmetry^55^. The ACT/graphene showed an increased *I*_D_/*I*_G_ ratio (∼1.21) compared with the ACT/graphite oxide with the *I*_D_/*I*_G_ ratio of ∼1.03. The increased *I*_D_/*I*_G_ ratio is ascribed to the re-establishment of graphene network during the reduction process, indicating smaller sizes, more defects and disordered structures of the graphene^14^. The XRD and Raman spectroscopy results jointly demonstrate successful removal of the oxygen-containing groups on the surfaces and edges of graphene oxide sheets during the reduction process.

### Cell performance

The as-prepared ACT/Co-Al LDH was used as the positive electrode, ACT/graphene as the negative electrode and the PVA-KOH gel as both the solid electrolyte and separator to construct a flexible asymmetric supercapacitor. The energy storage performances of the assembled cells and individual electrodes were studied by cyclic voltammetry (CV), galvanostatic (GV) charge/discharge and electrochemical impedance spectroscopy (EIS) in both two-electrode and three-electrode testing systems.

The three-electrode testing system was used to study the electrochemical reaction mechanisms of the as-prepared ACT, ACT/graphene and ACT/Co-Al LDH electrodes. The CV curves of the aforementioned electrodes at the scan rate of 25 mV s^−1^ with the potential windows ranging from −0.2 to 0.6 V are shown in Supplementary Fig. 2a. Both ACT and ACT/graphene electrodes showed quasi-rectangular-shaped CV curves, indicating ideal electrical double layer capacitive behaviour. The small peak at ∼0.25 V in the CV curve of ACT/graphene electrode is ascribed to the pseudo-capacitive reaction from the residual oxygen-containing groups on graphene sheets, resulting in a higher capacitance. The CV curve of the ACT/Co-Al LDH hybrid electrode showed a more complex shape with two pairs of redox peaks, which resulted from the typical Faradaic redox reactions of Co hydroxides, as described below^56^:


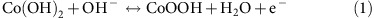



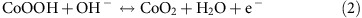


Supplementary Fig. 2b shows the GV charge/discharge performances of the ACT, ACT/graphene and ACT/Co-Al LDH electrodes at the current density of 2.5 A g^−1^. Compared with the triangular-shaped charge/discharge curves of the ACT and ACT/graphene electrodes, the ACT/Co-Al LDH electrode exhibited two charge/discharge voltage plateaus, which are the pseudocapacitive plateaus from the Co-Al LDH. The specific capacitance values of the ACT, ACT/graphene and ACT/Co-Al LDH electrodes were measured to be 90.1, 136 and 977.3 F g^−1^, respectively. The CV curves of the ACT/Co-Al LDH electrode at the scan rates of 25, 50, 75 and 100 mV s^−1^ in 6 M KOH electrolyte solution are shown in Supplementary Fig. 2c. All of them exhibited similar redox peaks, even at increased scan rates, indicating the quasi-reversible and continuous faradic redox reactions from Co-Al LDH that contributed remarkably to the pseudocapacitance. The GV charge/discharge curves of the ACT/Co-Al LDH electrode at different current densities are shown in Supplementary Fig. 2d. The specific capacitance values obtained from the ACT/Co-Al LDH at the current densities of 2.5, 5, 10 and 20 A g^−1^ are 977.3, 863, 727 and 545 F g^−1^, respectively, with a capacitance retention of ∼55.8% even when the current density increased from 2.5 to 20 A g^−1^.

**Figure 4:**
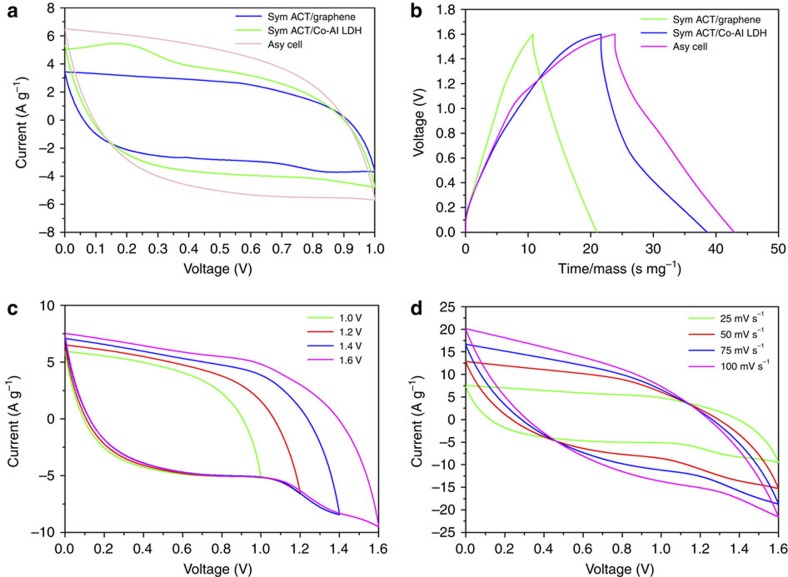
Electrochemical performance of symmetric and asymmetric cells. (**a**) CV curves of ACT/graphene//ACT/graphene and ACT/Co-Al LDH//ACT/Co-Al LDH symmetric cells as well as ACT/Co-Al LDH//ACT/graphene asymmetric cell with PVA/KOH polymer gel electrolyte in the voltage window of 1.0 V at a scan rate of 25 mV s^−1^. (**b**) Charge/discharge curves of ACT/graphene//ACT/graphene and ACT/Co-Al LDH//ACT/Co-Al LDH symmetric cells as well as ACT/Co-Al LDH//ACT/graphene asymmetric cell with PVA/KOH polymer gel electrolyte in the voltage window of 1.6 V at a current density of 12.5 mA cm^−2^ (*x* axis has been transformed to time/mass, based on the mass of the electrode). (**c**) CV curves of ACT/Co-Al LDH//ACT/graphene asymmetric cell with PVA/KOH polymer gel electrolyte under the voltage windows of 1, 1.2, 1.4 and 1.6 V at the scan rate of 25 mV s^−1^, respectively. (**d**) CV curves of ACT/Co-Al LDH//ACT/graphene asymmetric cell with PVA/KOH polymer gel electrolyte in the voltage windows of 1.6 V at different scan rates.

Figure 4a shows the CV curves of ACT/graphene//ACT/graphene and ACT/Co-Al LDH//ACT/Co-Al LDH symmetric cells as well as ACT/Co-Al LDH//ACT/graphene asymmetric cell with PVA/KOH polymer gel electrolyte at the scan rate of 25 mV s^−1^. The rectangular-shaped CV curves of the ACT/graphene symmetric cell suggest that the ACT/graphene mainly worked as an electrochemical double-layered capacitor. The broad peak at 0.25 V of the ACT/Co-Al LDH symmetric cell indicates the Faradic reaction from the battery-type Co-Al LDH. The CV area of the ACT/Co-Al LDH//ACT/graphene asymmetric cell was much larger than that of the ACT/graphene//ACT/graphene and ACT/Co-Al LDH//ACT/Co-Al LDH symmetric cells, suggesting the larger specific capacitance of ACT/Co-Al LDH//ACT/graphene asymmetric cell resulting from the high accessibility of electrolyte ions. Figure 4b shows the charge/discharge curves of the ACT/graphene//ACT/graphene and ACT/Co-Al LDH//ACT/Co-Al LDH symmetric cells as well as ACT/Co-Al LDH//ACT/graphene asymmetric cell. The ACT/graphene symmetric cell exhibited a triangular-shaped charge/discharge curve, implying an ideal capacitor character. Note that the ACT/Co-Al LDH symmetric cell exhibited a distorted charge/discharge profile, indicating the Faradic reaction during the charging/discharging process. The discharging capacities were measured to be 145.8, 131.75 and 77.9 F g^−1^ for the ACT/Co-Al LDH//ACT/graphene asymmetric cell, ACT/Co-Al LDH symmetric cell and ACT/graphene symmetric cell at the current density of 12.5 mA cm^−2^, respectively. Figure 4c shows the CV curves of the ACT/Co-Al LDH//ACT/graphene asymmetric cell with PVA/KOH polymer gel electrolyte in the respective voltage windows of 1.0, 1.2, 1.4 and 1.6 V at the scan rate of 25 mV s^−1^. The working voltage of the ACT/Co-Al LDH//ACT/graphene asymmetric cell can be extended to 1.6 V, which is essential for practical application. Figure 4d shows the CV curves of the ACT/Co-Al LDH//ACT/graphene asymmetric cell at different scan rates ranging from 25 to 100 mV s^−1^ at an operation window of 1.6 V. The current density increases with increasing scan rate. All the CV curves at different scan rates in Fig. 4e have similar shape, indicating stable reversibility and good rate performance of the ACT/Co-Al LDH//ACT/graphene asymmetric cell.

**Figure 5:**
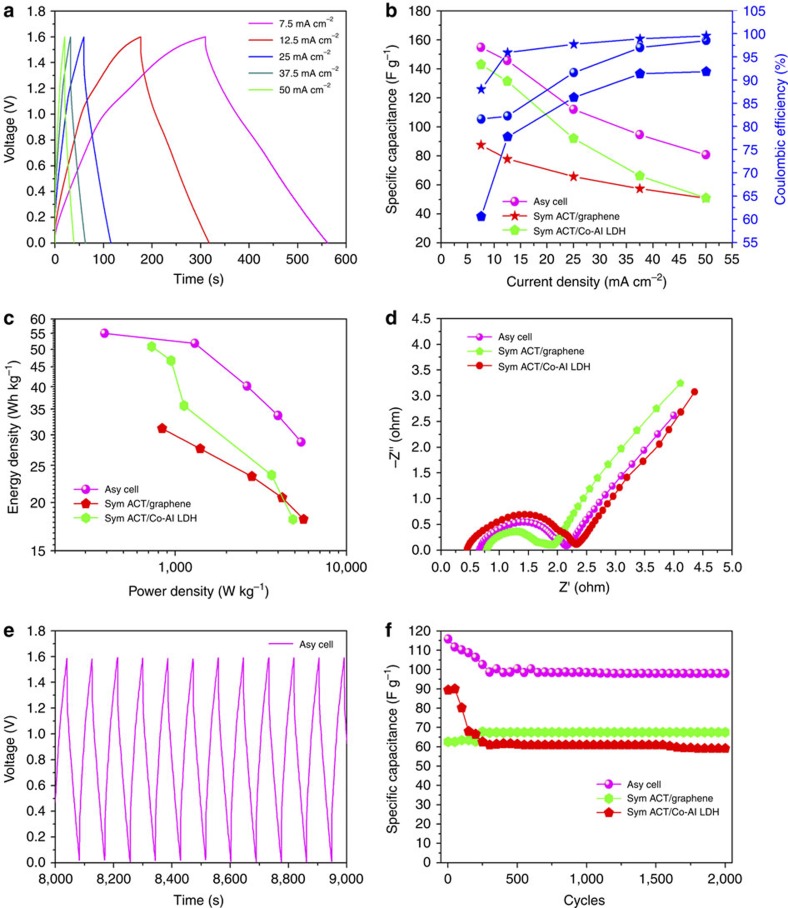
Electrochemical performance of the asymmetric cell. (**a**) Charge/discharge curves of ACT/Co-Al LDH//ACT/graphene asymmetric cell with PVA/KOH polymer gel electrolyte in the voltage window of 1.6 V at different current densities. (**b**) Specific capacitances and Coulombic efficiency of the ACT/graphene//ACT/graphene and ACT/Co-Al LDH//ACT/Co-Al LDH symmetric cells as well as ACT/Co-Al LDH//ACT/graphene asymmetric cell with PVA/KOH polymer gel electrolyte in the voltage window of 1.6 V. (**c**) Ragone plots of the ACT/graphene//ACT/graphene and ACT/Co-Al LDH//ACT/Co-Al LDH symmetric cells as well as ACT/Co-Al LDH//ACT/graphene asymmetric cell with PVA/KOH polymer gel electrolyte in the voltage window of 1.6 V. (**d**) Nyquist plots of the ACT/graphene//ACT/graphene and ACT/Co-Al LDH//ACT/Co-Al LDH symmetric cells as well as ACT/Co-Al LDH//ACT/graphene asymmetric cell with PVA/KOH polymer gel electrolyte in the voltage window of 1.6 V. (**e**) Representative charge/discharge curve of ACT/Co-Al LDH//ACT/graphene asymmetric cell with PVA/KOH polymer gel electrolyte in the voltage window of 1.6 V. (**f**) Cyclic performance of the ACT/graphene//ACT/graphene and ACT/Co-Al LDH//ACT/Co-Al LDH symmetric cells as well as ACT/Co-Al LDH//ACT/graphene asymmetric cell with PVA/KOH polymer gel electrolyte.

Rate performance and coulombic efficiency are important factors for the real power application of supercapacitors. Figure 5a shows the charge/discharge curves of ACT/Co-Al LDH//ACT/graphene asymmetric supercapacitor at different current densities. A high capacitance retention of 80.78 F g^−1^ was achieved even when the current density increased from 7.5 to 50 mA cm^−2^, indicating the excellent rate performance of the ACT/Co-Al LDH//ACT/graphene asymmetric cell. The rate performance and corresponding coulombic efficiency of ACT/graphene//ACT/graphene and ACT/Co-Al LDH//ACT/Co-Al LDH symmetric cells as well as ACT/Co-Al LDH//ACT/graphene asymmetric cell are comprehensively compared in Fig. 5b. The assembled ACT/graphene symmetric cell exhibited an excellent electrochemical stability (58% capacitance retention) and high coulombic efficiency (99.5% at 50 mA cm^−2^) in a wide range of current densities (7.5–50 mA cm^−2^). Whereas the ACT/Co-Al LDH symmetric cell showed poor rate performance (35% capacitance retention) and low coulombic efficiency (91.8% at 50 mA cm^−2^). Compared with the ACT/Co-Al LDH symmetric cell, the ACT/Co-Al LDH//ACT/graphene asymmetric cell exhibited not only improved capacitance retention (52.3% capacitance retention) but also enhanced coulombic efficiency (98.5% at 50 mA cm^−2^). At high charge/discharge rates the ions on electrode decreased rapidly with increasing rate, and the ions in the electrolyte diffused too slowly to satisfy the need of ions near the solid–liquid interface, leading to the decrease of capacitance^57^. The improvements in both rate capability and coulombic efficiency for the asymmetric cell result from the synergetic effects between the two distinct electrodes where the high conductive ACT/graphene with high rate capacity balanced the poor rate capacity of ACT/Co-Al LDH.

Energy density and power density are important factors for evaluating the practical application of supercapacitors. Figure 5c shows the Ragone plots of ACT/graphene//ACT/graphene and ACT/Co-Al LDH//ACT/Co-Al LDH symmetric cells as well as ACT/Co-Al LDH//ACT/graphene asymmetric cell. Compared with the symmetric cells, the ACT/Co-Al LDH//ACT/graphene asymmetric cell exhibited a higher energy density of 55.04 Wh kg^−1^ at the power density of 387.9 W kg^−1^ and maintained 28.72 Wh kg^−1^ at the power density of 5.44 kW kg^−1^. For an in-depth understanding of the electrochemical behaviour of the assembled symmetric and asymmetric cells, the EIS tests were carried out on the aforementioned cells (Fig. 5d). A similar shape can be found for all the impedance spectra, with a straight line at the low-frequency regime and an arc at the high-frequency region. The high-frequency arc is ascribed to the double-layer capacitance (*C*_dl_) and the charge transfer resistance (*R*_ct_) at the electrode and electrolyte interface, corresponding to the charge transfer-limiting process^58^. *R*_ct_ was directly measured from the diameter of the semicircle arc in the Niquist plot. Clearly, compared with the ACT/graphene (1.1 Ω) and ACT/Co-Al LDH (1.89 Ω) symmetric cells, the relatively smaller *R*_ct_ of the asymmetric cell resulted from the synergistic effect of the ACT/graphene and hierarchical ACT/Co-Al LDH architectures that facilitated the access of electrolyte ions to the active surface and shortened the ion diffusion path.

Cycling capability is another crucial requirement for the practical application of supercapacitors. The cyclic performances of ACT/graphene//ACT/graphene and ACT/Co-Al LDH//ACT/Co-Al LDH symmetric cells as well as ACT/Co-Al LDH//ACT/graphene asymmetric cell were evaluated at the current density of 12.5 mA cm^−2^ using a GV charge–discharge technique. An obvious capacity decay (∼35%) was observed for the ACT/Co-Al LDH symmetric cell, whereas there was a capacity increase (∼8%) for the ACT/graphene symmetric cell after 2,000 cycles. For the ACT/Co-Al LDH//ACT/graphene asymmetric cell, ∼16% decay of its original capacitance was noted after 2,000 cycles. The asymmetric cell exhibited linear and symmetrical characteristics in its charge/discharge curves, and no obvious ‘IR drop' was observed, indicating its low internal resistance (Fig. 5e). Compared with previously reported LDH-based asymmetric supercapacitors (see Table 1 for details), the electrochemical properties of the ACT/Co-Al LDH//ACT/graphene asymmetric cell, benefiting from the synergistic effects of ACT/graphene and flower-like ACT/Co-Al LDH, are highly competitive in terms of specific capacity, maximum energy and power densities, and cyclability. The assembled flexible asymmetric cell also worked well under a 180° folded state (Supplementary Fig. 3), a clear indication of the cell's excellent coupled mechanical and electrochemical robustness.

**Figure 6:**
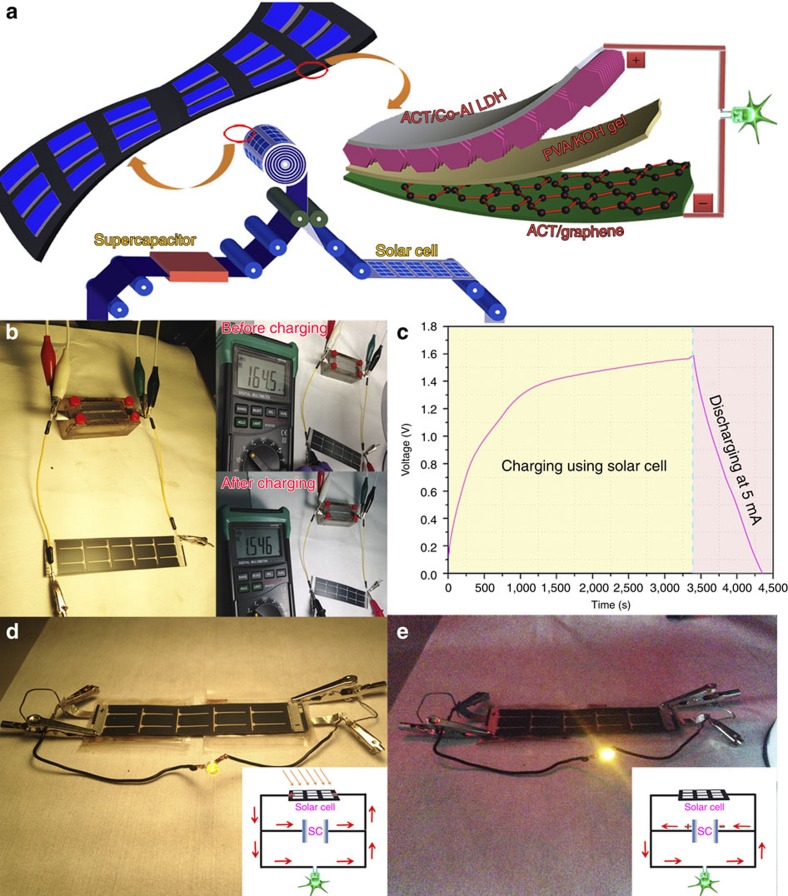
The charging/discharging processes of the assembled self-sustaining power pack. (**a**) Schematic illustration of the roll-to-roll manufacturing process for integrating a flexible solar cell with the supercapacitor into a self-sustaining power pack. (**b**) Digital photograph of the assembled solar cell/supercapacitor hybrid energy conversion and storage system, insets are the photographs of the open circuit potential of the ACT/Co-Al LDH//ACT/graphene asymmetric cell before charging and after charging with solar cell. (**c**) Charging curve of the ACT/Co-Al LDH//ACT/graphene asymmetric cell with solar cell, and discharging curve of the ACT/Co-Al LDH//ACT/graphene asymmetric cell at a current density of 5 mA cm^−2^. (**d**) Digital photograph of the assembled solar cell/supercapacitor hybrid power pack worked under the light, inset shows schematically the working circuit connection. (**e**) Digital photograph of the assembled solar cell/supercapacitor hybrid power pack worked without the light, inset shows schematically the working circuit connection.

Supercapacitors have been proven to be efficient and powerful energy storage devices to drive various electronic components. Furthermore, if a renewable energy source can be used to sustain an energy charge, the combined supercapacitor/energy source cell will provide continuous power for consumer electronics, forming a self-sustaining system without need for large, heavy batteries^59^. As the most sustainable and cleanest source of energy in the world, solar energy is usually limited by access to sunlight as restricted by time of day, location and weather. Combining solar cells with energy storage devices provides a promising solution to extend the practical applications of solar energy beyond the imposed restrictions of sunlight availability. As schematically illustrated in Fig. 6a, a flexible thin-film solar cell with an open circuit potential of 3 V under light was integrated with the flexible asymmetric cell to realize a combined energy conversion and storage system in a single device by a simple roll-to-roll process. Figure 6b illustrates the operation mechanism of the assembled solar cell/supercapacitor hybrid energy conversion and storage system. Insets of Fig. 6b are the photographs of the open circuit potential of the ACT/Co-Al LDH//ACT/graphene asymmetric supercapacitor before and after charging with the solar cell. Before charging by the solar cell, the open circuit potential of the asymmetric supercapacitor was 0.1645 V. Under the light source, the solar cell (with an open circuit potential of 3 V) serving as the power source charged the supercapacitor. After charging, the open circuit potential increased to 1.546 V. The corresponding charging (under light) and discharging curves (no light) of the asymmetric supercapacitor at the current density of 5 mA cm^−2^ were recorded by the electrochemical workstation (Fig. 6c), demonstrating the self-powered function of such hybrid energy conversion and storage system. Encouragingly, a high energy transfer efficiency of ∼43% was achieved from the solar cell to the capacitor. To further demonstrate the practical application of such integrated flexible energy system, a flexible solar cell was integrated with two flexible asymmetric cells connected in series by a facile roll-to-roll process, which holds a great promise for large-scale manufacturing of such hybrid cells (Fig. 6d,e). Insets of Fig. 6d,e illustrate the schematics of working circuit connection. Under light, the solar cell served as the power source to provide energy for the supercapacitor and LED. When the light was turned off, the stored energy in the supercapacitor in turn served as the power source for the LED, which enabled this hybrid energy system to work continuously for ∼10 min, overcoming the limitation of solar discontinuity. Such flexible, self-sustaining energy systems hold great potential for future portable/wearable electronics where they can reliably power devices as consumers move in and out of sunlight during their normal daily activities.

## Discussion

Two-dimensional battery-type Co-Al LDH nanosheets were anchored *in situ* on ACTs by a simple hydrothermal process. Separately, ACT fibres were wrapped with a highly conductive graphene coating by a simple dipping, drying and reducing process (ACT/graphene). A flexible, all-solid-state asymmetric supercapacitor (ACT/Co-Al LDH//ACT/graphene) was assembled using the nanostructured ACT/Co-Al LDH as the positive electrode, ACT/graphene as the negative electrode and PVA-KOH gel as both the solid-state electrolyte and separator. The hierarchical flower-like Co-Al LDH nanoarrays with interconnected nano-petals and pompon-like nano-stamens provided a highly open and porous scaffold-like structure, facilitating the transportation of electrolyte ions. In addition, the wrapped graphene on porous ACTs rendered a highly accessible surface area and good electrical conductivity, endowing the flexible asymmetric supercapacitor with higher rate performance. The assembled ACT/Co-Al LDH//ACT/graphene asymmetric supercapacitor exhibited an excellent combination of electrochemical and mechanical performance. Moreover, we demonstrated a low-cost, roll-to-roll manufacturing approach to combine the flexible asymmetric supercapacitor with a flexible solar cell to build an integrated, self-sustaining power pack, which is scalable for industrial manufacturing. Importantly, such hybrid energy storage devices could continuously power a commercial LED, demonstrating a great potential for the future of self-powered nanotechnology.

## Methods

### Preparation of flexible ACT/Co-Al LDH positive electrode

All of the chemicals were used after purchasing without further purification. A piece of commercial cotton T-shirt was first cleaned using distilled water in an ultrasonic bath before activation. Activation of the cotton T-shirt was performed as described in detail in our previous report^47^. First, a piece of cotton T-shirt was dipped into 1 M NaF solution and soaked for 1 h. The wet textile was then dried at 120 °C for 3 h. Second, the NaF-treated cotton textile was transferred into a horizontal tube furnace and kept at 1,000 °C for 1 h with a continuous argon gas flow (300 s.c.c.m.). After cooling down to room temperature, the as-obtained ACTs were washed with distilled water to remove the residual NaF and then dried at 80 °C for 6 h. Co-Al LDH nanoarrays were grown *in situ* on ACT fibres via a simple hydrothermal process. Briefly, 0.582 g of Co(NO_3_)_2_·6H_2_O, 0.518 g of Al(NO_3_)_3_·6H_2_O, 0.296 g of NH_4_F and 0.6 g of urea were dissolved in 36 ml distilled water. The resulting solution was transferred into a 50-ml Teflon-lined stainless autoclave with a piece of vertically suspended ACT (1 × 2 cm^2^) in the solution. Then, the autoclave was placed in an electric oven at 100 °C for 24 h. Finally, the as-prepared products were washed thoroughly with ethanol and distilled water and dried at 80 °C overnight to produce ACT/Co-Al LDH composite.

### Preparation of flexible ACT/graphene-negative electrode

Graphite oxide was synthesized by exfoliating natural graphite flakes using a modified Hummers method^60^. The obtained graphite oxide solution was further exfoliated by ultrasonication in an ultrasonic bath for 1 h to prepare graphene oxide. Then, the above graphene oxide solution was centrifuged at 3,000 r.p.m. for 5 min to remove residual aggregates, forming a brown aqueous colloid with a concentration of ∼4 mg ml^−1^. A piece of ACT (1 × 2 cm^2^) was then soaked with the graphene oxide aqueous colloid. After drying at 80 °C for 6 h, the as-prepared ACT with graphene oxide coating was heated at 450 °C for 1 h with argon/hydrogen mixture gas (v/v, 90/10) to reduce flexible ACT/graphene oxide to ACT/graphene-negative electrode.

### Characterization methods

The morphology and microstructure of the as-prepared products were characterized by SEM (FEI Quanta 650), TEM (JEOL 2000FX), HRTEM (FEI Titan), and atomic force microscopy (Nanoscope IIIa). The crystallographic structure of the synthesized materials was determined by a PANalytical X'Pert Pro Multi-Purpose Diffractometer equipped with Cu *K*_α_ radiation (*λ*=0.15406, nm). The Raman spectra of the ACT/graphene were recorded by a Renishaw InVia Raman microscope at 785 nm. Surface areas of the active materials were measured by physical adsorption of N_2_ at 77 K (Quantachrome Autosorb iQ surface area and pore size analyser) and calculated by the BET method.

### Fabrication and characterization of integrated flexible self-sustaining power pack

The electrochemical performances of the prepared ACT, ACT/graphene and ACT/Co-Al LDH electrodes were characterized by both three-electrode and two-electrode systems. The three-electrode tests were carried out in 6 M KOH aqueous electrolyte at room temperature. The prepared ACT, ACT/graphene and ACT/Co-Al LDH were used as the working electrodes, platinum foil (1 × 1 cm^2^) and a saturated calomel electrode were used as the counter and reference electrodes, respectively. The mass of the ACT, ACT/graphene and ACT/Co-Al LDH was measured to be 2, 3.45 and 4.1 mg cm^−2^, respectively. The asymmetric supercapacitor used in this paper was assembled with two pieces of flexible, face-to-face electrodes (ACT/Co-Al LDH-positive and ACT/graphene-negative) separated by the solid-state, polymer PVA/KOH gel electrolyte (ACT/Co-Al LDH//ACT/graphene). The polymer gel electrolyte was prepared by mixing 3 g KOH and 6 g PVA in 60 ml deionized water by magnetically stirring the solution at 80 °C until the solution became clear. Then, the above solution was transferred into a flat Petri-dish to naturally solidify to PVA/KOH gel film electrolyte. Two pieces of flexible electrodes separated by the PVA/KOH gel film were used to assemble the flexible asymmetric cell. The as-obtained solid-state, polymer PVA/KOH gel film severed as both the separator and electrolyte. The electrochemical properties of the assembled flexible asymmetric cell were measured using a CHI 660E electrochemical workstation. CV, GV charge/discharge curves and EIS in the frequency range from 100 kHz to 0.05 Hz with an AC perturbation of 5 mV were used to evaluate the electrochemical performance of the flexible solid-state asymmetric supercapacitors. The integrated flexible energy conversion and storage unit was assembled by combining a piece of commercial flexible solar panel (with an open circuit potential of 3 V and energy efficiency of 22%, from PowerFilm) with an energy storage unit consisting of two pieces of asymmetric cell connected in series using an electrical rolling machine (MSK-HRP-MR100A, MTI). Two pieces of assembled asymmetric flexible supercapacitors were connected in series and then coated with double-side adhesive tapes. At the same time, the bottom surface of the flexible solar cell was also coated with double-side adhesive tapes. As illustrated in Fig. 6a, the flexible supercapacitor and the flexible solar cell were integrated together by the roll-to-roll rolling press machine. During the roll-to-roll fabrication process, the top surface of the solar cell (collecting sunlight) was protected by using the soften cloth to obtain a flexible, solar cell/supercapacitor, self-sustaining power pack. The open circuit potential and charge/discharge processes were monitored by the CHI 660E electrochemical workstation.

### Calculation

Specific capacitances derived from GV tests were calculated from equation 1 as follows:


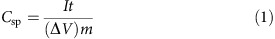


where *C*_sp_ (F/g), *I* (A), *t* (s), *m* (g) and Δ*V* (V) are the specific capacitance, the discharge current, the discharge time, the mass of the active material and the total potential window, respectively.

Energy density (*E*) and power (*P*) density derived from GV tests were calculated from the following equations:


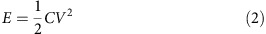



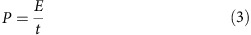


where *E* (Wh/kg), *C* (F/g), *V* (V), *P* (W/kg) and *t* (s) are the energy density, specific capacitance, potential window, power density and discharge time, respectively.

## Additional information

**How to cite this article:** Gao, Z. *et al.* Cotton-textile-enabled flexible self-sustaining power packs via roll-to-roll fabrication. *Nat. Commun.* 7:11586 doi: 10.1038/ncomms11586 (2016).

## Supplementary Material

Supplementary Information Supplementary Figures 1-3.

## Figures and Tables

**Table 1:** Comparison of the as-prepared Co-Al LDH asymmetric supercapacitor with previously published results.

**Reference**	**Electrode materials**	**Specific capacitance (F** **g**^**−1**^**)**	**Working potential (V)**	**Maximum energy density (Wh** **kg**^**−1**^**)**	**Maximum Power density (kW** **kg**^**−1**^**)**	**Cyclic performance (retention)**
24	Ni-Co LDH//rGO	550	1.6	188	7.32	82% after 5,000 cycles
56	GSP/Co-Al LDH//SGC	—	1.6	41.2	9.3	84% after 2,000 cycles
61	Ni-Al LDH@CNPs//AC	138	1.6	47.7	51	88.9% after 2,000 cycles
62	Ni-Co LDH//AC	125.2	1.2	23.7	5.82	92.7% after 5,000 cycles
63	Co–Al LDHs–CNTs//AC	80.6	1.6	28	∼6	88.9% after 1,000 cycles
64	Ni-Co LDH//AC	—	1.5	25.3	10.5	91.2% after 10,000 cycles
65	NiCo-LDHs@CNT/NF//APDC/NF	210.9	1.8	89.7	8.7	78% after 1,200 cycles
Our work	Co-Al LDH/ACT//ACT/graphene	145.8	1.6	55.04	5.4	87.54% after 2,000 cycles

AC, activated carbon; CNPs, carbon nanoparticles; GSP, integrated porous Co–Al hydroxide nanosheets; LDH, layered double hydroxide; NF, nickel foam; rGO, reduced graphene oxide; SGC, sandwiched graphene/porous carbon.
