# Supplementary material for: Cotton-textile-enabled flexible self-sustaining power packs via roll-to-roll fabrication
Source: Nat Commun. 2016 May 18;7:11586. doi: 10.1038/ncomms11586 (PMC4873971; doi:10.1038/ncomms11586)
Supplement: Supplementary Information — Supplementary Figures 1-3. [file ncomms11586-s1.pdf]

Supplementary Figures

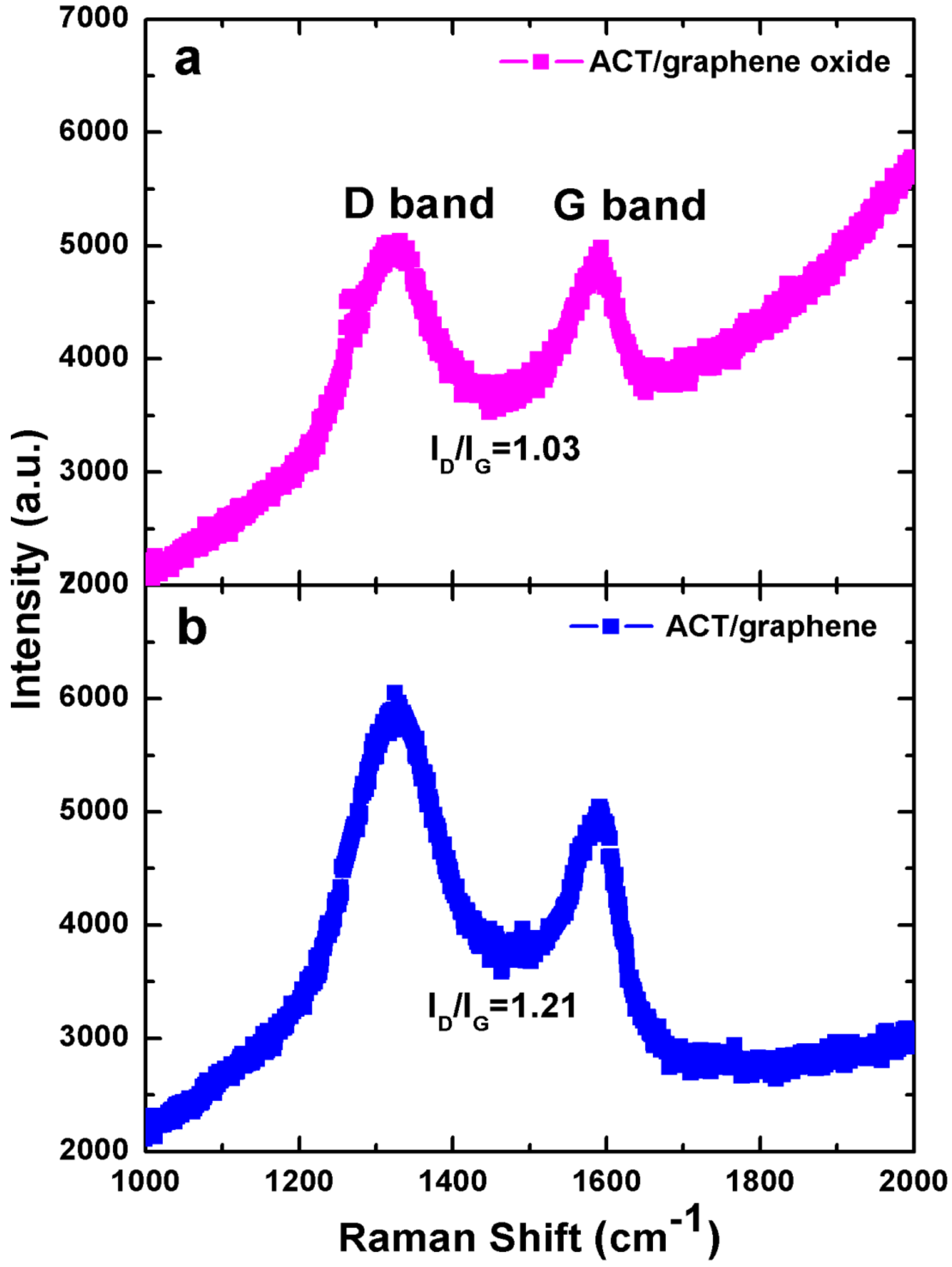

Supplementary Figure 1: Raman characterization. Raman spectra of ACT/graphene oxide (**a**) and ACT/graphene (**b**) composites.

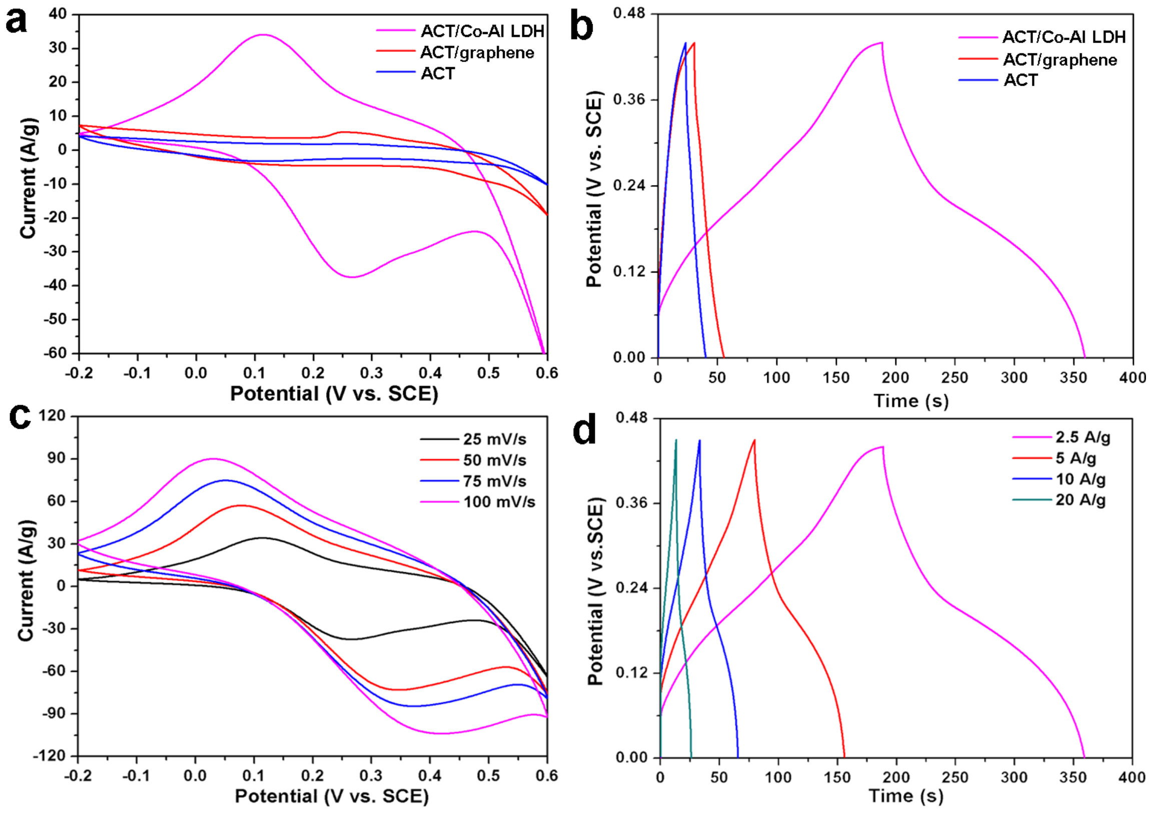

Supplementary Figure 2: Electrochemical performance test by three-electrode system. (**a**) CV curves of the ACT, ACT/graphene, and ACT/Co-Al LDH electrodes with the 6 M KOH electrolyte in the potential window of -0.2-0.6 V at a scan rate of 25 mV s^{-1}. (**b**) Charge/discharge curves of the ACT, ACT/graphene, and ACT/Co-Al LDH electrodes with the 6 M KOH electrolyte in the potential window of 0-0.44 V at a current density of 2.5 A g^{-1}. (**c**) CV curves of ACT/Co-Al LDH electrode with the 6 M KOH electrolyte in the potential windows of -0.2 – 0.6 V at different scan rates. (**d**) Charge/discharge curves of ACT/Co-Al LDH electrode with the 6 M KOH electrolyte in the potential windows of 0 – 0.44 V at different current densities.

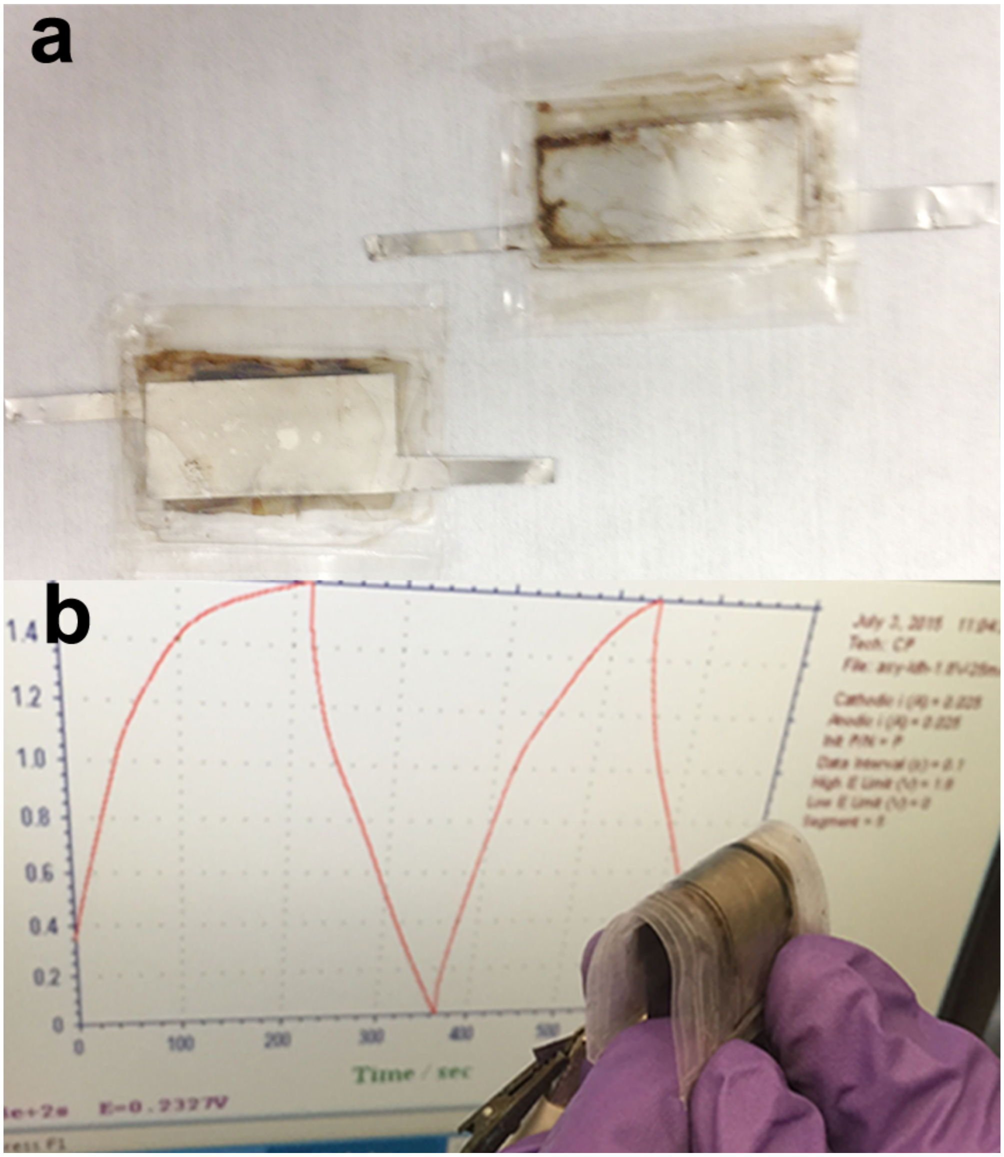

Supplementary Figure 3: Digital photographs of the assembled flexible asymmetric cell. (**a**) Digital photograph of the assembled flexible asymmetric cell under normal state. (**b**) Digital photograph of the assembled flexible asymmetric cell under folded state, demonstrating its excellent flexibility.
